# Application of a Genetically Encoded Biosensor for Live Cell Imaging of L-Valine Production in Pyruvate Dehydrogenase Complex-Deficient *Corynebacterium glutamicum* Strains

**DOI:** 10.1371/journal.pone.0085731

**Published:** 2014-01-17

**Authors:** Nurije Mustafi, Alexander Grünberger, Regina Mahr, Stefan Helfrich, Katharina Nöh, Bastian Blombach, Dietrich Kohlheyer, Julia Frunzke

**Affiliations:** 1 IBG-1: Biotechnology, Forschungszentrum Jülich, Jülich, Germany; 2 Institute of Biochemical Engineering, University of Stuttgart, Stuttgart, Germany; Centre National de la Recherche Scientifique - Université de Toulouse, France

## Abstract

The majority of biotechnologically relevant metabolites do not impart a conspicuous phenotype to the producing cell. Consequently, the analysis of microbial metabolite production is still dominated by bulk techniques, which may obscure significant variation at the single-cell level. In this study, we have applied the recently developed Lrp-biosensor for monitoring of amino acid production in single cells of gradually engineered L-valine producing *Corynebacterium glutamicum* strains based on the pyruvate dehydrogenase complex-deficient (PDHC) strain *C. glutamicum* Δ*aceE*. Online monitoring of the sensor output (eYFP fluorescence) during batch cultivation proved the sensor's suitability for visualizing different production levels. In the following, we conducted live cell imaging studies on *C. glutamicum* sensor strains using microfluidic chip devices. As expected, the sensor output was higher in microcolonies of high-yield producers in comparison to the basic strain *C. glutamicum ΔaceE*. Microfluidic cultivation in minimal medium revealed a typical Gaussian distribution of single cell fluorescence during the production phase. Remarkably, low amounts of complex nutrients completely changed the observed phenotypic pattern of all strains, resulting in a phenotypic split of the population. Whereas some cells stopped growing and initiated L-valine production, others continued to grow or showed a delayed transition to production. Depending on the cultivation conditions, a considerable fraction of non-fluorescent cells was observed, suggesting a loss of metabolic activity. These studies demonstrate that genetically encoded biosensors are a valuable tool for monitoring single cell productivity and to study the phenotypic pattern of microbial production strains.

## Introduction

In natural environments, inherent cell-to-cell variation within isogenic populations and resulting formation of subpopulations often bears an overall fitness advantage for the whole population [1]. Variation of phenotypic traits has been reported to promote the division of labor or as “bet-hedging” strategy to enable rapid adaption to sudden environmental changes [1], [2], [3], [4]. In biotechnological processes, however, arising phenotypic variation and the formation of insufficiently producing subpopulations can adversely affect the entire production process [5], [6]. Besides its biological origin, heterogeneity within large scale cultivation processes is caused by environmental variations at the micro scale, e.g., of dissolved gases, pH and nutrients caused by insufficient mixing or the formation of biofilms [7], [8], [9], [10].

Nowadays, bioprocess monitoring is still dominated by bulk approaches delivering average values for the whole population. Masking of cell-to-cell variation might consequently result in misleading interpretations of biological phenomena [1]. Analysis of growth and product formation of single microbial cells would provide a detailed insight into the phenotypic structure of the population representing a further, important step towards a systems level understanding of microbial processes. A major drawback of this approach is, however, the limited number of microbially produced metabolites which confer an observable phenotype to the respective cell; among the few exceptions are natural chromophores, such as carotenoids. This challenge demands the development of novel tools and techniques for single-cell quantification and real-time monitoring of inconspicuous, small metabolites [11], [12], [13]. In this context, genetically encoded biosensors capable of detecting small molecules inside the cell and transforming this information into an optical readout (e.g. fluorescence signal) represent a powerful tool for single-cell analysis of microbial production strains. The implementation of genetically encoded metabolite sensors in live cell imaging studies performed in microfluidic cultivation systems offers the advantage of long-term observation of single-cell growth and metabolite production with high spatial and temporal resolution [14], [15].

*Corynebacterium glutamicum* represents one of the most important platform organisms in industrial biotechnology; dominating the global, large-scale production of amino acids (e.g., L-glutamate, L-lysine, and L-valine) [16]. Recent studies using multiparameter flow cytometry revealed phenotypic heterogeneity in terms of viability, membrane potential and growth activity of *C. glutamicum* wild type cells grown in shake flasks [17]. However, population heterogeneity during production processes has not been studied in detail for this species, yet. Recently, our group reported on the development of a genetically encoded metabolite sensor (Lrp-sensor), which enables the cytosolic detection of branched-chain amino acids or L-methionine in single *C. glutamicum* cells. The sensor is based on the transcriptional regulator Lrp of *C. glutamicum* which activates expression of the *brnFE* operon, encoding an amino acid export system, upon accumulation of the effector amino acids L-methionine, L-leucine, L-isoleucine, and L-valine [18], [19], [20], [21]. In previous studies, the sensor was successfully applied in flow cytometry-based high-throughput (HT) screenings for the isolation of mutants producing amino acids and in first live cell imaging studies of the L-valine production strain *C. glutamicum ΔaceE* [22].

*C. glutamicum* was successfully engineered for efficient L-valine production within the last years [23], [24], [25], [26], [27]. Strains are based on the deletion of the *aceE* gene, which encodes the E1p subunit of the pyruvate dehydrogenase complex (PDHC) and an additional plasmid-based overexpression of the *ilvBNCE* genes encoding L-valine biosynthesis enzymes. The resulting strain *C. glutamicum ΔaceE* (pJC4-ilvBNCE) was further improved by additional deletion of the genes encoding pyruvate:quinone oxidoreductase (*pqo*), phosphoglucose isomerase (*pgi*), and pyruvate carboxylase (*pyc*). These modifications led to a series of strains, based on the same parental strain (*C. glutamicum ΔaceE*), with a stepwise increasing product yield (Y*_P/S_*) reaching the theoretical maximal Y*_P/S_* of 0.86 mol L-valine *per* mol of glucose in *C. glutamicum ΔaceE Δpqo Δpgi Δpyc* (pJC4-ilvBNCE) [28]. The common and characteristic feature of these PDHC-deficient strains is the onset of the production phase only after a complete consumption of the acetate which is required for growth [23]. Due to these properties the strains represent an ideal testing ground for biosensor performance. In this work, we have successfully applied the genetically encoded Lrp-sensor for live cell imaging studies to monitor amino acid production, growth, and viability in *C. glutamicum* L-valine production strains in a time-resolved manner and at single cell resolution.

## Materials and Methods

### Bacterial strains, media, and growth conditions

Bacterial strains and plasmids used or constructed in this work are listed in Table 1. Unless stated otherwise, pre-cultures of *C. glutamicum* were inoculated with single colonies from a fresh brain heart infusion (BHI) agar plate containing 51 mM acetate and incubated in 4 ml BHI complex medium with 51 mM acetate for 6 h at 30°C and 170 rpm. This first pre-culture was used to inoculate a 100 ml shake flask containing 20 ml CGXII minimal medium [29] with 222 mM glucose and 154 mM acetate. The cells of the second pre-culture were cultivated overnight at 30°C and 120 rpm, washed twice with 0.9% (w/v) saline and then used to inoculate the main culture to an optical density (OD_600_) of 1. If not stated differently, cells in the main culture were cultivated under the same conditions as in the pre-culture. Potassium acetate was used in all experiments performed in this study. *Escherichia coli* DH5α was grown aerobically in LB medium on a rotary shaker (120 rpm) or on LB agar plates at 37°C [30]. Where appropriate, the media contained kanamycin (25 µg ml^−1^ for *C. glutamicum* or 50 µg ml^−1^ for *E. coli* DH5α) or isopropyl β-_D_-1-thiogalactopyranoside (IPTG), as indicated. For online monitoring of growth and fluorescence, cells were cultivated in 48-well flowerplates using the BioLector system (m2p-labs GmbH, Aachen, Germany) [31]. Cultivation conditions have been described previously [22].

**Table 1:** Bacterial strains, plasmids, and oligonucleotides used in this study.

Strains, plasmids	Relevant characteristics	Reference
**Strains**		
*E. coli DH5α*	*supE44, ΔlacU169* (*φ*80*lacZ*DM15), *hsdR17, recA1, endA1, gyrA96,thi1,relA1*.	Invitrogen
*C. glutamicum* ATCC13032	Biotin-auxotrophic wild type.	[48]
*ΔaceE*	*C. glutamicum* wild type with deletion of the *aceE* gene, coding for the E1p subunit of the pyruvate dehydrogenase-complex (PDHC).	[49]
Δ*aceE* Δ*pqo*	*C. glutamicum* Δ*aceE* strain with deletion of the *pqo* gene, coding for pyruvate:quinone oxidoreductase.	[50]
Δ*aceE* Δ*pqo* Δ*pgi*	*C. glutamicum* Δ*aceE* Δ*pqo* strain with deletion of the *pgi* gene, coding for the phosphoglucose isomerase.	[28]
ΔaceE Δ*pqo* Δ*pgi* Δ*pyc*	*C. glutamicum* Δ*aceE* Δ*pqo* Δ*pgi* strain with deletion of the *pyc* gene, coding for the pyruvate carboxylase.	[28]
*C. glutamicum* sensor strain	*C. glutamicum* wild type strain with chromosomally integrated Lrp-sensor (integrated into the intergenic region of cg1121-cg1122) and pJC4-ilvBNCE-crimson plasmid.	This work.
ΔaceE sensor strain	ΔaceE strain with chromosomally integrated Lrp-sensor (cg1121-cg1122) and pJC4-ilvBNCE-crimson plasmid.	This work.
ΔaceE Δ*pqo* sensor strain	ΔaceE Δ*pqo* strain with chromosomally integrated Lrp-sensor (cg1121-cg1122) and pJC4-ilvBNCE-crimson plasmid.	This work.
ΔaceE Δ*pqo* Δ*pgi* sensor strain	ΔaceE Δ*pqo* Δ*pgi* strain chromosomally integrated Lrp-sensor (cg1121-cg1122) and pJC4-ilvBNCE-crimson plasmid.	This work.
ΔaceE Δ*pqo* Δ*pgi* Δ*pyc* sensor strain	ΔaceE Δ*pqo* Δ*pgi* Δ*pyc* strain with chromosomally integrated Lrp-sensor (cg1121-cg1122) and pJC4-ilvBNCE-crimson plasmid.	This work.
**Plasmids**		
pJC1	*E. coli-C. glutamicum* shuttle vector, Kan^R^, *oriV_Ec_, oriV_Cg_*.	[51]
pJC1-lrp-brnF'-eyfp	pJC1derivative containing Lrp-sensor cassette, which consists of *lrp* (cg0313), the intergenic region of *lrp brnF* (cg0314) and a transcriptional fusion of *brnF* with *eyfp*.	[22]
pJC4-ilvBNCE	pJC1derivative carrying the *ilvBNCE* genes coding for the L-valine biosynthetic enzymes acetohydroxyacid synthase, isomeroreductase, and transaminase B.	[25]
pJC4-ilvBNCE-crimson	pJC4-ilvBNCE derivative containing *e2-crimson* under transcriptional control of P*_tac_*.	This work.
pK18-mobsacB	Vector for allelic exchange in *C. glutamicum*; Kan^R^; *oriV_Ec_, sacB, lacZα*.	[52]
pK18-mobsacB-cg1121, cg1122-Lrp-sensor	pK18mobsacB derivative for genomic integration of the Lrp-sensor in the intergenic region of cg1121-cg1122 in *C. glutamicum*.	This work.
**Oligonucleotides**	**Sequence (5′ → 3′)**	
lacI-fw	TCAAGCCTTCGTCACTGGTCC	This work.
E2-Crimson-rv	CTACTGGAACAGGTGGTGGCG	This work.
Int-cg1121-fw	TTGGCGTGTGGTTGGTTAG	This work.
Int-cg1122-rv	CGCATCAAGCAGATCTCTG	This work.

### Recombinant DNA work

Standard methods like PCR, DNA restriction or ligation were carried out according to standard protocols [30]. Synthesis of oligonucleotides and sequencing analysis were performed by Eurofins MWG Operon (Ebersfeld, Germany). The vector pE2-Crimson was derived by Clontech Laboratories (Mountain View, CA, USA). For the construction of pJC4-ilvBNCE-crimson, *e2-crimson* under transcriptional control of P*_tac_* was amplified using oligonucleotides lacI-fw and E2-Crimson-rv [32]. The PCR product was cloned into the vector pJC4-ilvBNCE [25] using the Bst1107I restriction site. For chromosomal integration of the Lrp-sensor, the sensor cassette was inserted into the intergenic region of cg1121-cg1122 using pK18-mobsacB-cg1121, cg1122 [22]. The transfer of the integration plasmid into *C. glutamicum* and selection of the first and second recombination events were performed as described previously [33]. Correct integration at the chromosomal locus was verified by colony PCR using primers Int-cg1121-fw and Int-cg1122-rv.

### Quantification of amino acids

For determination of amino acid concentrations in the supernatant, samples of the cultures were centrifuged (13,000 rpm, 10 min, 4°C) and amino acid concentration was quantified by reversed-phase high-pressure liquid chromatography as described before [22].

### Microfluidic chip cultivation

Microfluidic PDMS-glass chips were fabricated according to [14], [34]. The microfluidic monolayer cultivation system utilized in the present study was designed for microcolony growth and growth-coupled phenotypic studies at the single-cell level [14], [35]. The device features 100 arrays of monolayer cultivation chambers (1 µm×40 µm×40 µm; height × width × length) for HT monitoring of microcolony growth under constant environmental conditions. The microfluidic chip connected to 1 ml disposable syringes (Omnifix 40 Duo, B. Braun Melsungen AG, Germany) for continuous media supply was placed inside an in-house manufactured incubator for temperature and atmosphere control. Media flow was controlled with syringe pumps (neMESYS, Cetoni GmbH, Korbussen, Germany). The incubator was mounted onto a fully motorized inverted Nikon Eclipse Ti microscope (Nikon GmbH, Düsseldorf, Germany) suitable for time-lapse live cell imaging. The setup was equipped with a focus assistant (Nikon PFS) compensating for thermal drift during long-term microscopy and a CFI Plan Apo Lambda DM 100X-magnification, 1.45 numeric aperture oil phase contrast objective. Temperature control of the objective was realized using an objective heater (ALA OBJ-Heater, Ala Scientific Instruments, USA). A cell suspension of OD_600_ 0.5-1, transferred from a pre-culture at exponential growth phase, was infused to the system. After successful cell seeding, the growth medium was infused at approximately 100 nl min^−1^ per channel.

### Live cell imaging and image analysis

The microscope was equipped with an ANDOR LUCA R DL604 EMCCD camera (Andor Technology plc., Belfast, UK) for image recording and a 300 W Xenon light source for fluorescence excitation (Lambda DG4, Sutter Instruments, USA). Following fluorescence filters (AHF Analysentechnik, Germany) were applied: i) YFP: HQ 500/20 (excitation filter), Q515 (dichroic), and HQ 535/30 (emission); ii) E2-Crimson: HQ 600/37 (excitation filter), Q630 (dichroic) and Q675/67 (emission). Phase contrast and fluorescence microscopy images of several microcolonies were captured in 15 min time intervals. Growth and fluorescence were recorded for 10–20 isogenic microcolonies during each experiment. Image analysis was performed with the Nikon NIS Elements AR software package. The visualization of lineage tree was realized using our in-house developed Python-based software.

## Results

### Online monitoring of L-valine production

**Figure 1:**
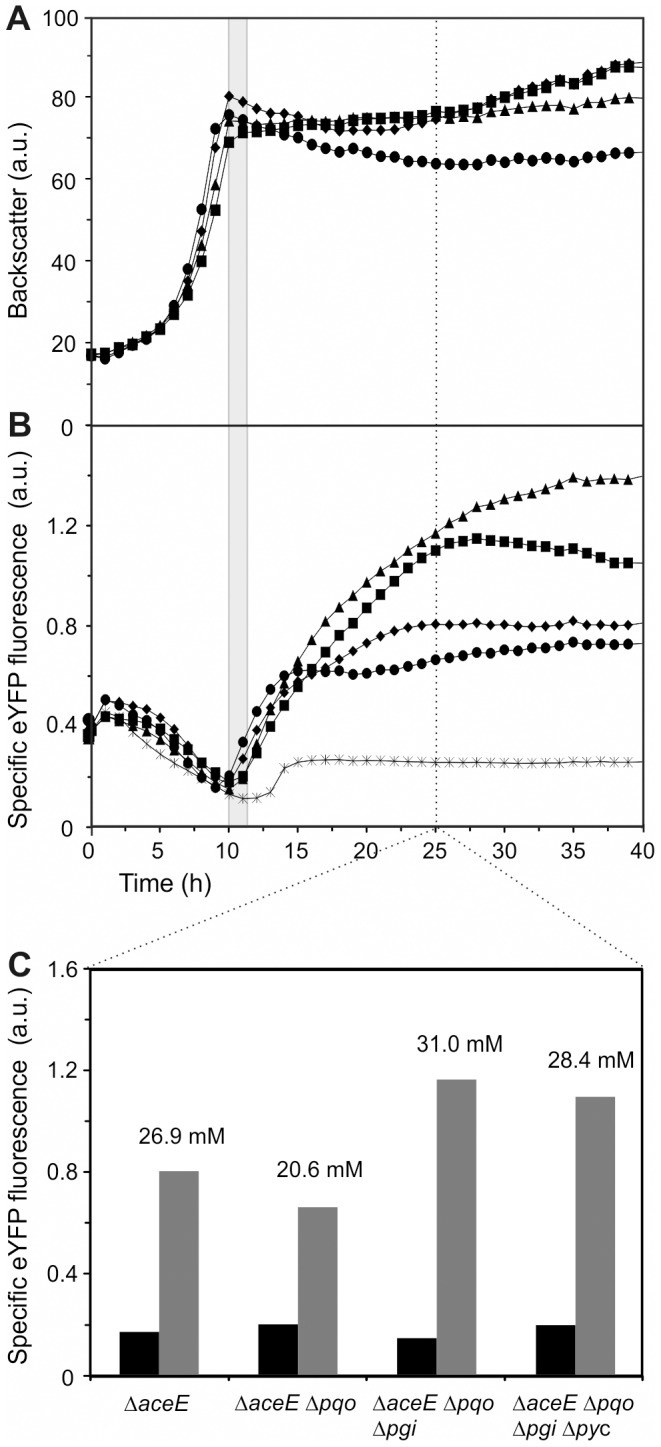
Biosensor-based online monitoring of L-valine production in PDHC-deficient *C. glutamicum* strains. (**A**) Growth and (**B**) Lrp-sensor output (eYFP fluorescence) of the sensor strains *C. glutamicum* ATCC 13032 wild type (stars), *ΔaceE* (diamonds), *ΔaceE Δpqo* (circles), *ΔaceE Δpqo Δpgi* (triangles), and *ΔaceE Δpqo Δpgi Δpyc* (squares) cultivated in CGXII minimal medium containing 222 mM glucose and 154 mM acetate. Data represent average values of three independent cultivations. The transition of the producer strains into the stationary and production phase is highlighted by the grey area. (**C**) EYFP fluorescence of respective strains at the beginning of the production phase (black bars) and twelve hours after the initiation of L-valine production (grey bars). L-valine concentration (mM) in the supernatant of the respective strain 25 h after beginning of cultivation as measured by HPLC is indicated above the grey bars.

Previously, we presented the Lrp-biosensor as a convenient tool to discriminate between low levels of L-valine production and wild type level [22]. In the present study, we assessed the performance of the biosensor to monitor the course of L-valine production over time in high-yield and basic *C. glutamicum* L-valine production strains [28]. For this purpose, the Lrp-sensor was chromosomally integrated into the different strains in order to avoid plasmid-based effects, such as a fluctuating copy number or plasmid loss. Strains under study were *ΔaceE*::Lrp-sensor (pJC4-ilvBNCE-crimson), *ΔaceE Δpqo*::Lrp-sensor (pJC4-ilvBNCE-crimson), *ΔaceE Δpqo Δpgi*::Lrp-sensor (pJC4-ilvBNCE-crimson), and *ΔaceE Δpqo Δpgi Δpyc*::Lrp-sensor (pJC4-ilvBNCE-crimson), henceforth referred to as “sensor strains” (Table 1). The sensor strains as well as the wild type *C. glutamicum* ATCC 13032 (pJC4-ilvBNCE-crimson) containing the Lrp-sensor were cultivated in CGXII minimal medium supplied with 154 mM acetate and 222 mM glucose in microtiter plates (0.75 ml filling volume) in the BioLector cultivation system, enabling online measurement of biomass (backscatter) and eYFP fluorescence [31]. Within the first 10 to 12 hours, the strains grew exponentially while exhibiting a decrease in fluorescence over time (Figure 1A, B). This residual fluorescence and the decrease of the signal in the first hours were also observed in the wild type strain and therefore can likely be ascribed to changes of auto- or background fluorescence during growth. After depletion of the acetate required for growth, the cells entered the stationary phase and an increase in eYFP fluorescence was detected, indicating growth-decoupled L-valine production of the strains (Figure 1B). At the early production phase all strains exhibited a similar sensor signal, but split up in the course of the cultivation. Twelve hours after shifting into the production phase an almost twofold higher sensor output of the high-yield producers (*ΔaceE Δpqo Δpgi* and *ΔaceE Δpqo Δpgi Δpyc* sensor strains) was observed in comparison to the basic producers (*ΔaceE* and *ΔaceE Δpqo* sensor strains) (Figure 1B, C). The fluorescence of the *ΔaceE* and the *ΔaceE Δpqo* sensor strains reached its maximum intensity within five to ten hours, suggesting constant internal L-valine concentrations. In contrast, the fluorescence of the *ΔaceE Δpqo Δpgi* and the *ΔaceE Δpqo Δpgi Δpyc* sensor strains increased for about 15 hours, reflecting the higher potential for L-valine production of these strains. Determination of amino acid concentration in the supernatant confirmed different levels of L-valine production, ranging from 30 mM L-valine in average for the *ΔaceE Δpqo Δpgi* sensor strain and the *ΔaceE Δpqo Δpgi Δpyc* sensor strain to 23 mM in average for the *ΔaceE* sensor strain and the *ΔaceE Δpqo* sensor strain (Figure 1C). These results demonstrate that the Lrp-sensor does not only provide an ON/OFF response (wild type *versus* production strain), but can be applied for online monitoring of production processes in basic as well as high-yield production strains, since (i) information about initiation of the production process is provided, (ii) the course of metabolite production process is displayed over time, and (iii) different levels of productivity are revealed.

### Live cell imaging of L-valine production

**Figure 2:**
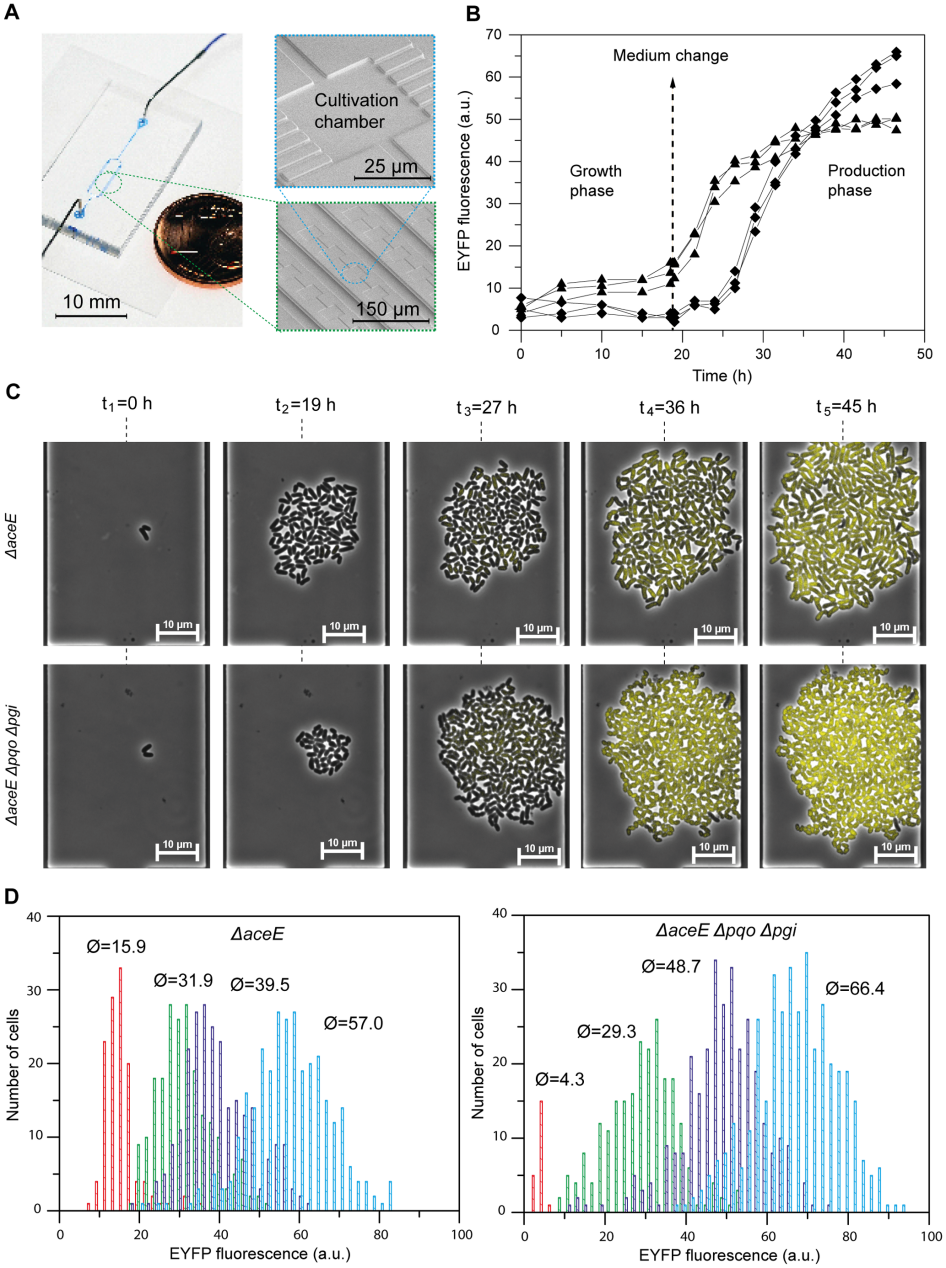
Live cell imaging of L-valine production strains using microfluidic monolayer cultivation chambers . (**A**) Illustration of the microfluidic cultivation chambers. The system consists of several arrays of picoliter sized monolayer cultivation chambers. (**B**) Fluorescence emission of three entire microcolonies (average eYFP signal *per* colony area) of the *ΔaceE* sensor strain (triangles) and the *ΔaceE Δpqo Δpgi* sensor strain (diamonds) over time. Fluorescence was measured every 2.5 h. (**C**) Growth (t_1_–t_2_) and production phase (t_3_–t_5_) of isogenic microcolonies of the *ΔaceE* sensor strain (upper row) and the *ΔaceE Δpqo Δpgi* sensor strain (lower row). (**D**) Histograms illustrating fluorescence distribution within a representative microcolony of the *ΔaceE* sensor strain (left) and the *ΔaceE Δpqo Δpgi* sensor strain (right). The eYFP signal of single cells was measured at t = 19 h (red), t = 26 h (green), t = 34 h (purple), and t = 46 h (blue). Average fluorescence values are indicated above the respective peaks. All cultivations were performed in microfluidic chambers shown in (A) in CGXII minimal medium containing 154 mM acetate and 222 mM glucose during growth phase or CGXII with 222 mM glucose during the production phase, respectively.

In the following experiments, we analyzed the applicability of the Lrp-biosensor in live cell imaging studies to investigate growth, physiology, and metabolic activity of single cells in a time-resolved manner. For this purpose, *C. glutamicum* L-valine production stains were cultivated in monolayer microfluidic cultivation chambers under constant environmental conditions (Figure 2A) [14]. After single-cell inoculation into microfluidic chambers, cells were grown in CGXII medium with 154 mM acetate and 222 mM glucose as carbon source. A medium change (after 18.5 hours) to CGXII medium containing 222 mM glucose initiated L-valine production. Figure 2 shows two representative colonies of the *ΔaceE* sensor strain and the *ΔaceE Δpqo Δpgi* sensor strain during growth (t_1_, t_2_) and L-valine production phase (t_3_–t_5_) (Video S1, S2). Cells gradually stopped growing and simultaneously exhibited progressively increasing eYFP fluorescence after the medium switch.

The average fluorescence signal of three microcolonies (fluorescence signal *per* colony area) of the *ΔaceE* sensor strain and the *ΔaceE Δpqo Δpgi* sensor strain during growth and production phase is depicted in Figure 2B. In contrast to the *ΔaceE Δpqo Δpgi* sensor strain, colonies of the *ΔaceE* sensor strain already displayed a low eYFP signal during the growth phase. Although starting at different levels, the fluorescence of both strains increased comparably when L-valine production was initiated. In agreement with the results obtained from microtiter plate cultivations (see Figure 1), colonies of the high-yield producer *ΔaceE Δpqo Δpgi* sensor strain showed an overall higher final fluorescence in comparison to colonies of the *ΔaceE* sensor strain. This was also reflected by the single-cell fluorescence of the respective strains during the production phase (Figure 2D). Measurement of single-cell fluorescence of both strains revealed a broadening Gaussian distribution in the course of the experiment.

### Correlation of sensor output and metabolic activity

**Figure 3:**
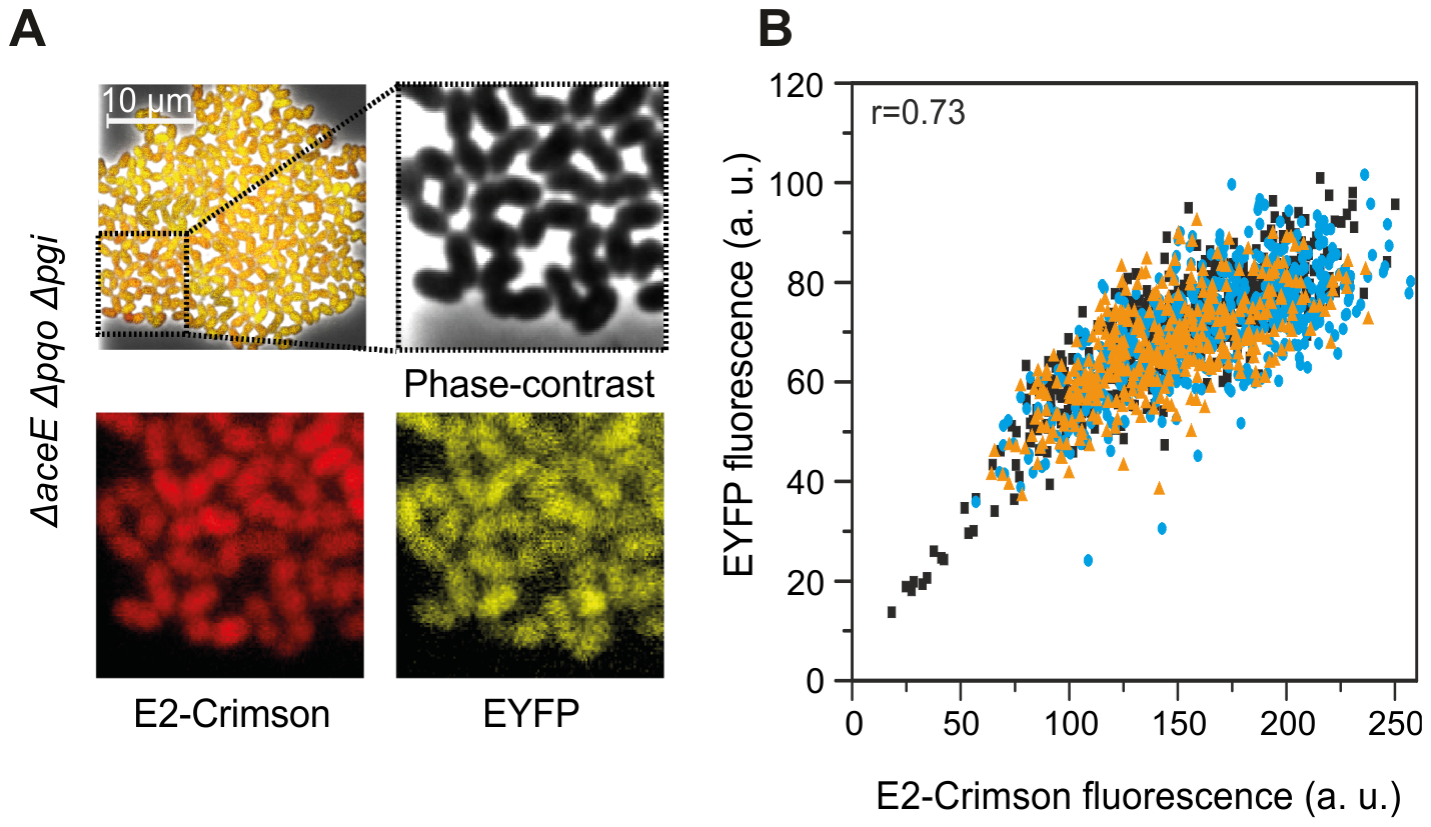
Correlation of the Lrp-sensor output (eYFP) and the plasmid marker E2-Crimson. (**A**) Microscopy overlay plot of phase-contrast, eYFP and E2-Crimson signal of an isogenic microcolony of the *Δace*E *Δpqo Δpgi* sensor strain after 46 h (see Figure 2C). (**B**) Dot plot displaying eYFP and E2-Crimson signal of single cells of three isogenic microcolonies (triangles, circles, and diamonds) of the *Δace*E *Δpqo Δpgi* sensor strain.

In further studies, we intended to validate the correlation of the Lrp-biosensor output and the physiological state of the respective cells. In other words, is a low sensor output an indication for a reduced metabolic activity or even death of the respective cell? To introduce a measure for plasmid stability in L-valine producing sensor strains, *e2-crimson* coding for far-red fluorescence protein E2-Crimson was placed under control of P*_tac_* in the vector pJC4-ilvBNCE containing the gene cluster *ilvBNCE* for overexpression of the L-valine biosynthesis genes [32]. The vector with the integrated plasmid marker was transferred into the *ΔaceE Δpqo Δpgi* sensor strain and eYFP (Lrp-biosensor) and E2-Crimson (plasmid marker) fluorescence emission were recorded for microcolonies grown in microfluidic cultivation chambers. Figure 3A shows a representative colony of the *ΔaceE Δpqo Δpgi* sensor strain at the end of the production phase (∼46 h). Single-cell eYFP and E2-Crimson fluorescence were recorded and are depicted in a correlation plot, with each dot representing a single cell (Figure 3B). Overall, a strong correlation between eYFP and E2-Crimson signal was observed (r = 0.73±0.09, n = 1436). Only a minor amount of cells (<1%) displayed a high E2-Crimson, but low eYFP signal. Metabolically inactive or dead cells (low signal for eYFP and E2-Crimson) were rarely detected. Nevertheless, in a few cases we also observed microcolonies with an increased number of non-fluorescent cells (see section 3.4.). Their frequency, however, strongly depended on the chosen colony and cell density during cultivation.

### Occurrence of non-fluorescent cells during the production phase

During the production phase, we always observed the occurrence of some non-fluorescent cells. Live cell imaging studies enable the investigation of this phenomenon in a time-resolved manner and the discrimination between different types of non-fluorescent cells, e.g. lysed, dead cells or dormant forms which resume growth after a while.

**Figure 4:**
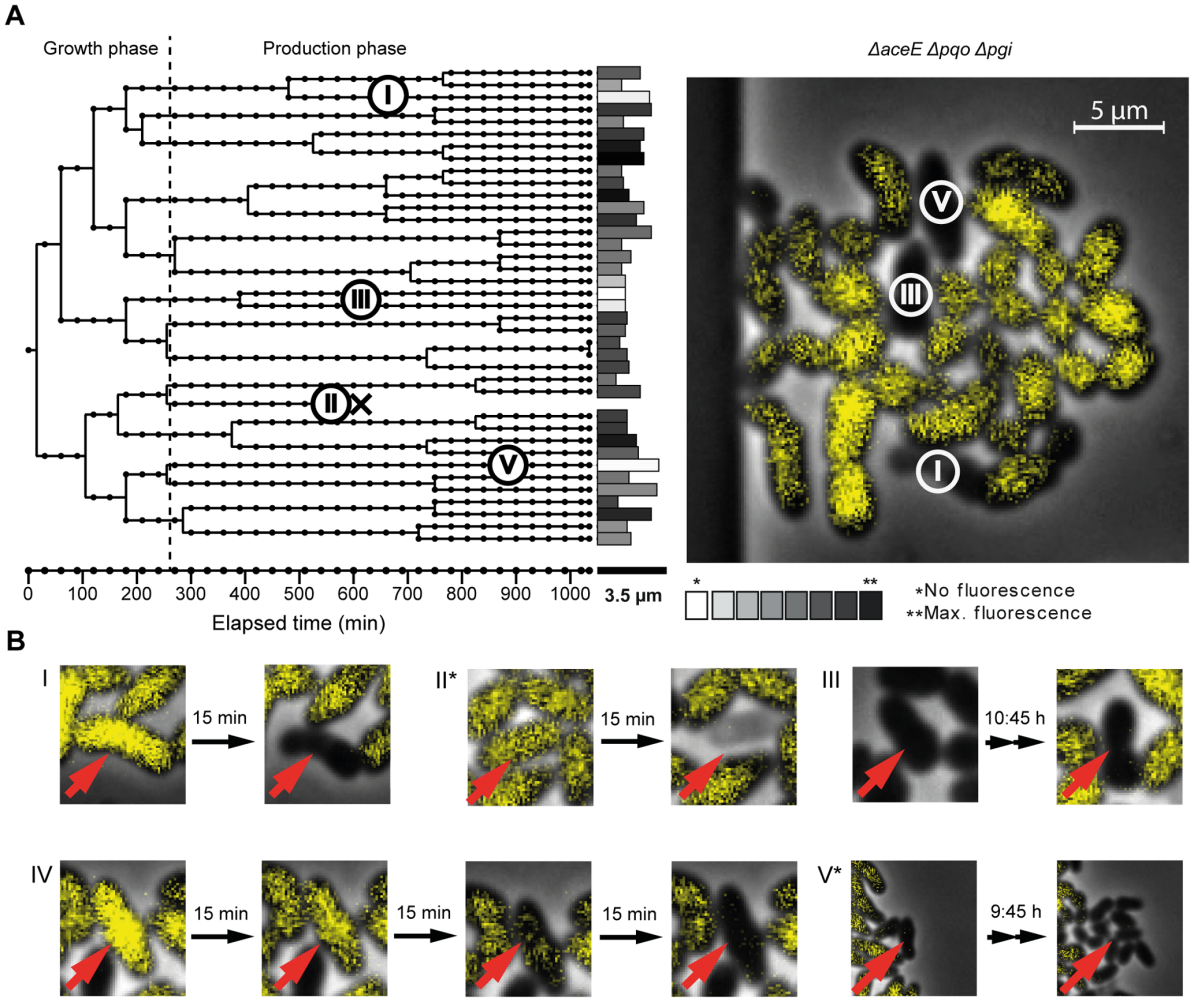
Occurrence of non-fluorescent cells during the production phase. (**A**) Microcolony and lineage tree of the *ΔaceE Δpqo Δpgi* sensor strain. Different types of non-fluorescent cells are illustrated in B. (**B**) **(I+II)** Lysing cells and **(III)** dormant/or dead cell, which do not switch from growth to production. **(IV)** Leaky cell that shows decreasing fluorescence signal over time, potentially caused by a permeabilized cell membrane. (V^*^) Cells showing slow growth, but no production. Images marked with an asterisk show cells of another microcolony of the *ΔaceE Δpqo Δpgi* sensor strain, not shown in this figure.

Under conditions described in section 3.2, we usually observed colonies showing a typical Gaussian distribution of eYFP intensity, but occasionally colonies with an increased number of non-fluorescing cells were found (<1% of cells) (Figure 4). We tracked different cells in several microcolonies, clustered them and found different types of non-producing cells (Figure 4, Video S3). The majority of cells, however, underwent transition from growth to production and will not be discussed here. The first type of non-producing cells initiated L-valine production, but showed a sudden cell lysis at a later time (Figure 4; I, II and IV). These cells were rarely seen at constant environmental conditions, but were more frequently observed when colony growth exceeded the chamber size, leading to densely packed colonies. A second fraction of cells neither initiated L-valine production nor showed growth after a change of medium. These cells might either represent dead or dormant cells (Figure 4, III). Finally, we also observed non-producing cells which did not enter production phase, but continued to grow (Figure 4, V), although no carbon source, i.e. acetate, was provided in the medium. One possible explanation would be that these cells have adapted their growth to utilizing glucose as carbon source.

### Phenotypic heterogeneity of *C. glutamicum* L-valine production strains

In contrast to growth in well-controlled microfluidic devices, cells in typical shake flask or bioreactor cultivations face significant fluctuations with respect to metabolite accumulation and physical parameters (pH, O_2_, etc.). Remarkably, when we exchanged the minimal medium used in the abovementioned studies against a non-defined complex medium (CGXII containing low amounts of BHI), we observed a strong impact on the phenotypic pattern with respect to amino acid production in all strains analyzed.

**Figure 5:**
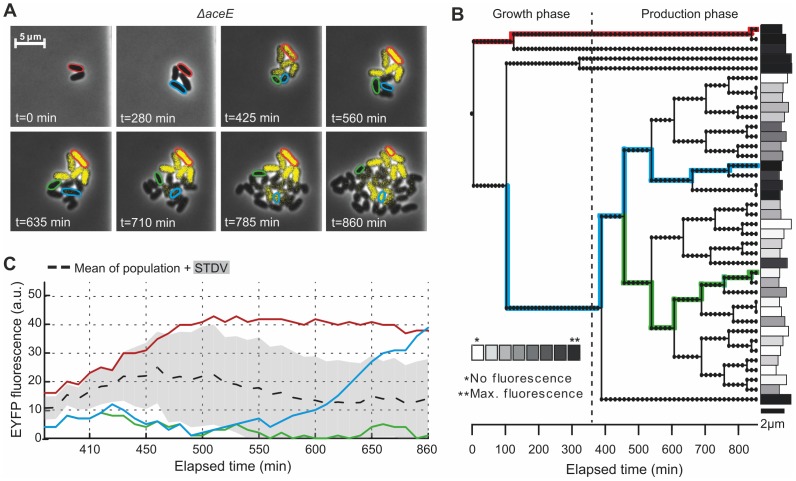
Biosensor-driven analysis of phenotypic heterogeneity. In the presence of low amounts of complex carbon sources, significant cell-to-cell variability in the switch from growth to L-valine production was observed. (**A**) Growth and production phase (initiated after 340 min) of an isogenic microcolony of the *ΔaceE* sensor strain and (**B**) the lineage tree of the respective microcolony highlighting several single cell traces. EYFP fluorescence was quantified in single cells after 860 min. (**C**) Single cell traces of fluorescence output of marked cells (see A and B) and average emission of the whole colony (black, dashed line, SD  =  grey shading). Cultivation was performed in CGXII minimal medium containing 154 mM acetate, 222 mM glucose and 0.5% BHI during growth phase or 222 mM glucose and 0.5% BHI during production phase, respectively.

Starting from a single cell, growth of an isogenic microcolony of the *ΔaceE* sensor strain in CGXII medium with 154 mM acetate, 222 mM glucose and 0.5% BHI was monitored in the microfluidic cultivation system. After a primary growth phase, cells were supplemented with 222 mM glucose and 0.5% BHI to trigger L-valine production. Although most of the cells switched from growth to production (Figure 5A, Video S4), in approximately 50% of the recorded colonies one or several single cells continued growing after the medium change. This observed bistability in the decision of switching from growth to production was observed for all L-valine producing strains under study when the cells were grown in the presence of low amounts of BHI (Figure S1 in File S1, Video S5). It was also observed in cells without the Lrp-sensor, which indicates that the observed split in phenotypes is not caused by the sensor itself (Figure S2 in File S1). Figure 5 shows two exemplary cells, originating from the same mother cell, where one cell (red) divided for the last time and initiated L-valine production directly after the medium switch, while the sister cell continued to grow (t_D_ = 105±21 min, n = 5). However, some of the descendants later also switched to L-valine production (blue) or continued growth throughout the course of the experiment (green). This experiment illustrated that, depending on the cultivation conditions, recording of the average fluorescent output of the whole population would mask the significant variation at the single-cell level (Figure 5C). In contrast, a uniform switching behavior was observed when only glucose was present in the production medium illustrating the strong impact of slight changes in medium composition on the phenotypic structure of a particular population.

## Discussion

Nowadays, multiple physiological parameters in single cells can be analyzed by flow cytometry or fluorescence microscopy using a constantly increasing number of fluorescent dyes and staining protocols [36]. Single-cell productivity is a key performance indicator in biotechnological production processes, which is unfortunately often masked by typical bulk-based analysis. However, technological limitations and a lack of convenient tools for accurate single-cell analysis, prevented in-depth analysis of productivity in microbial bioprocesses. In the present work, the genetically encoded Lrp-biosensor is introduced as a powerful tool for single cell analysis of production strains.

The Lrp sensor cassette was inserted into the genome of different *C. glutamicum* L-valine producing strains in order to avoid the effect of plasmid copy number fluctuations on the sensor output. In first proof of principle experiments, the genomically integrated metabolite sensor proved suitable to visualize different levels of L-valine production in gradually engineered strains [28]. Thus, the sensor is not restricted to provide an ON/OFF response (WT *versus* production strain), but reflects more subtle differences with respect to the metabolic activity of single cells. Remarkably, almost all eYFP-negative cells (>95%) of the L-valine producing sensor strains showed no growth when sorted on agar plates FACS and stained positive for PI, indicating an impaired membrane integrity (data not shown). This is further supported by the high correlation of sensor output (eYFP) and the signal of the integrated plasmid marker (E2-Crimson) shown in Figure 3A, B. Similar correlations were recently described for studies based on a GFP-sensor enabling the detection of carbon-limited conditions in *E. coli* as well as for a reporter system in yeast, in which the expression of *gfp* was set under control of a ribosomal protein promoter [37], [38].

*C. glutamicum* L-valine production strains were analyzed by live cell imaging during growth inside microfluidic chip devices to investigate the phenotypic pattern at the single-cell level [14]. The sensor output of isogenic colonies of the different production strains showed a broad Gaussian distribution suggesting phenotypic heterogeneity with respect to L-valine production. The peak width increased proportional to the mean fluorescence of the populations, which is also observed when fluorescent reporter genes are set under control of inducible promoters, such as P*_tac_* (data not shown). This pattern was obtained in all strains under study and no difference was observed in strains with increased precursor availability (Δ*pqo*, Δ*pcx*) or increased NADPH supply (Δ*pgi*) suggesting that these factors have no impact on the peak width of the sensor output. However, several parameters may influence the distribution of the reporter output of sensor strains, including cofactor supply of the involved biosynthetic enzymes, cell cycle, carbon source uptake or even stochastic effects on gene expression [5].

Individual cell tracking by time-lapse microscopy revealed different types of non-producing cells besides the productive main population. Sudden and progressive cell lysis and cells in a dormant state were observed. Additionally, a complete lack of the switch from growth to production phase was observed, with cells continuing growth at a low rate. These cells might utilize carbon sources, which are low concentrated, but are continuously supplied by the medium flow. For example, protocatechuic acid, required as iron-chelator, can be used as carbon source by *C. glutamicum* [39], [40]. Alternatively, cellular metabolism might have adapted to overflow of glucose, bypassing the reactions catalyzed by the PDH complex (deletion of *aceE*) and the pyruvate:quinone oxidoreductase (*pqo*) and providing acetyl-CoA as precursor for TCA-cycle. Studies of Litsanov *et al.* described that in spite of deleting the genes for the known acetate-synthesizing pathways in *C. glutamicum*, residual acetate formation during cultivation in minimal medium was still observed in strains engineered for aerobic succinate production [41].

In the presence of low amounts of complex medium, we observed a bistability in the cells decision to initiate L-valine production or to continue growth (Figure 5, Figure S1 and S3 in File S1, Video S4, S5). Besides the expected transition from growth to production, cells either showed unhampered growth and cell division or a time-delayed switch to production (Figure S3 in File S1). In larger-scale production processes the occurrence of such subpopulations may have a major impact on process efficiency, as these cells might overgrow the entire population in the course of time and, consequently, resources are depleted for biomass formation instead of being converted to the final product. These biosensor-based analysis, again, demonstrate that despite targeted, genetic manipulation on cells to work as efficient, uniform microbial factories, phenotypic variation might lead to the occurrence of drastic differences in cellular productivity even under the well-controlled cultivation conditions present in microfluidic chambers. Continued growth of some cells might be based on different energetic states of the cells or differences in the level of transporter proteins. A low amount of a specific permease is sufficient to induce an autocatalytic positive feedback resulting in a non-uniform induction behavior, as described for lactose uptake and catabolism by Novick and Weiner more than 50 years ago or by Siegele *et al.* for expression driven from the *araBAD* promoter [42], [43], [44].

## Conclusions

In this study, we describe the amino acid sensing Lrp-sensor as a valuable, non-invasive tool to monitor the metabolic activity of PDHC-deficient *C. glutamicum* L-valine producers at single-cell resolution. In recent years, a number of metabolite sensors based on RNA aptamers or transcription factors were reported, increasing the number of accessible metabolites [12], [45], [46], [47]. Future studies will aim at unraveling the underlying molecular mechanisms of the observed phenotypic variation, benchmarking this approach for the analysis and improvement of strains and biotechnological production processes.

## Supporting Information

File S1 This file ncludes Figures S1, S2 and S3. **Figure S1, Phenotypic heterogeneity of the** ***ΔaceE Δpqo Δpgi Δpyc*** **sensor strain** **upon switch from growth to production phase.** **(A)** Microcolony showing transition to producing cells or **(B)** a mixture of growing and producing cells after medium switch (initiated after 240 min). In approximately 50% of the recorded colonies one or several single cells continued growth after medium switch. **(C, D)** Fluorescence histograms depicting single cell fluorescence to selected times during growth (0–240 min) and production phase (0–1200 min) of the microcolonies shown in A **(C)** and B **(D)**. Cultivation was performed in CGXII minimal medium containing 154 mM acetate, 222 mM glucose and 0.5% BHI during growth phase or 222 mM glucose and 0.5% BHI during production phase, respectively. **Figure S2, Phenotypic heterogeneity of** ***ΔaceE*** **and** ***ΔaceE Δpqo Δpgi*** **upon switch from growth to production phase.** **(A)** ***Δ*** *aceE* microcolonies where all cells stopped growth (blue stars) upon transition to the production phase (upper row) or a mixture of growing (red stars) and non-growing cells (lower row) after initiation of the production phase. In approximately 50% of the recorded colonies one or several single cells continued growth after medium switch (initiated after 250 min). **(C)** *ΔaceE Δpqo Δpgi* microcolonies. In the upper row, all cells stopped growth whereas in the lower row a microcolony is shown were some cells continued growth after initiation of the production phase. In approximately 50% of the recorded colonies one or several single cells continued growth after medium switch (initiated after 250 min). These findings confirm that the phenotypic split shown in Figure 5 is not due to the presence of the Lrp-sensor. Cultivation was performed in CGXII minimal medium containing 154 mM acetate, 222 mM glucose and 0.5% BHI during growth phase or 222 mM glucose and 0.5% BHI during production phase, respectively. **Figure S3, Single cell traces of the** ***ΔaceE Δpqo Δpgi Δpyc*** **sensor strain upon switch from growth to production phase. (A)** Single cell traces showing the switch from growth (cell length  =  blue line) to production (fluorescence  =  squares) after several cell divisions during production phase (t = 8.5 h, t = 15.0 h). **(B)** Single cell traces showing no switch from growth to production. Single cell traces are taken from the cultivation of *ΔaceE Δpqo Δpgi Δpyc* sensor strain shown in Figure S1.
(PDF)

Video S1 **Growth and production of** ***C. glutamicum*** **ATCC 13032** ***ΔaceE*** **sensor strain.** Upon the switch to the production phase, cells gradually stopped growing and simultaneously exhibited progressively increasing eYFP fluorescence. Growth phase: CGXII medium with 154 mM acetate and 222 mM glucose; production phase: CGXII medium with 222 mM glucose as carbon source.
(WMV)

Video S2 **Growth and production of** ***C. glutamicum*** **ATCC 13032** ***ΔaceE Δpqo Δpgi*** **sensor strain.** Upon the switch to the production phase, cells gradually stopped growing and simultaneously exhibited progressively increasing eYFP fluorescence. Growth phase: CGXII medium with 154 mM acetate and 222 mM glucose; production phase: CGXII medium with 222 mM glucose as carbon source.
(WMV)

Video S3 **Occurrence of non-fluorescent cells during the production phase of the** ***C. glutamicum*** **ATCC 13032** ***ΔaceE Δpqo Δpgi*** **sensor strain.**
(WMV)

Video S4 **Phenotypic heterogeneity of the** ***C. glutamicum ΔaceE*** **sensor strain.** Cells were grown in CGXII medium with 154 mM acetate, 222 mM glucose and 0.5% BHI. After a primary growth phase, cells were supplemented with 222 mM glucose and 0.5% BHI to trigger L-valine production. Although most of the cells switched from growth to production (Figure 5A), in approximately 50% of the recorded colonies one or several single cells continued growing after the medium change.
(WMV)

Video S5 **Microcolonies of the** ***C. glutamicum ΔaceE Δpqo Δpgi*** **sensor strain displaying phenotypic heterogeneity.** Growth of six microcolonies (CGXII medium with 154 mM acetate, 222 mM glucose and 0.5% BHI) is shown in microfluidic chip devices. After a primary growth phase, cells were supplemented with 222 mM glucose and 0.5% BHI to trigger L-valine production. In the upper three colonies all cells showed a switch from growth to production. In the lower three colonies one or several single cells continued growing after initiation of the production phase.
(WMV)
