# Supplementary material for: Application of a Genetically Encoded Biosensor for Live Cell Imaging of L-Valine Production in Pyruvate Dehydrogenase Complex-Deficient Corynebacterium glutamicum Strains
Source: PLoS One. 2014 Jan 17;9(1):e85731. doi: 10.1371/journal.pone.0085731 (PMC3895001; doi:10.1371/journal.pone.0085731)
Supplement: File S1 — This file ncludes Figures S1, S2 and S3. Figure S1, Phenotypic heterogeneity of the ΔaceE Δpqo Δpgi Δpyc sensor strain upon switch from growth to production phase. (A) Microcolony showing transition to producing cells or (B) a mixture of growing and producing cells after medium switch (initiated after 240 min). In approximately 50% of the recorded colonies one or several single cells continued growth after medium switch. (C, D) Fluorescence histograms depicting single cell fluorescence to selected times during growth (0–240 min) and production phase (0–1200 min) of the microcolonies shown in A (C) and B (D). Cultivation was performed in CGXII minimal medium containing 154 mM acetate, 222 mM glucose and 0.5% BHI during growth phase or 222 mM glucose and 0.5% BHI during production phase, respectively. Figure S2, Phenotypic heterogeneity of ΔaceE and ΔaceE Δpqo Δpgi upon switch from growth to production phase. (A) Δ aceE microcolonies where all cells stopped growth (blue stars) upon transition to the production phase (upper row) or a mixture of growing (red stars) and non-growing cells (lower row) after initiation of the production phase. In approximately 50% of the recorded colonies one or several single cells continued growth after medium switch (initiated after 250 min). (C) ΔaceE Δpqo Δpgi microcolonies. In the upper row, all cells stopped growth whereas in the lower row a microcolony is shown were some cells continued growth after initiation of the production phase. In approximately 50% of the recorded colonies one or several single cells continued growth after medium switch (initiated after 250 min). These findings confirm that the phenotypic split shown in Figure 5 is not due to the presence of the Lrp-sensor. Cultivation was performed in CGXII minimal medium containing 154 mM acetate, 222 mM glucose and 0.5% BHI during growth phase or 222 mM glucose and 0.5% BHI during production phase, respectively. Figure S3, Single cell traces of the ΔaceE Δpqo Δpgi Δpyc sen [file pone.0085731.s001.pdf]

Supplemental material

Application of a genetically encoded biosensor for live cell imaging of L-valine production in pyruvate dehydrogenase complex-deficient *Corynebacterium glutamicum* strains

Nurije Mustafi[1§], Alexander Grünberger[1§], Regina Mahr[1], Stefan Helfrich[1], Katharina Nöh[1], Bastian Blombach[2], Dietrich Kohlheyer[1], and Julia Frunzke[1]*

[1]IBG-1: Biotechnology, Forschungszentrum Jülich GmbH (Research Centre Jülich), 52425 Jülich, Germany

[2]Institute of Biochemical Engineering, University of Stuttgart, 70569 Stuttgart, Germany

[$] Both authors contributed equally to this work.

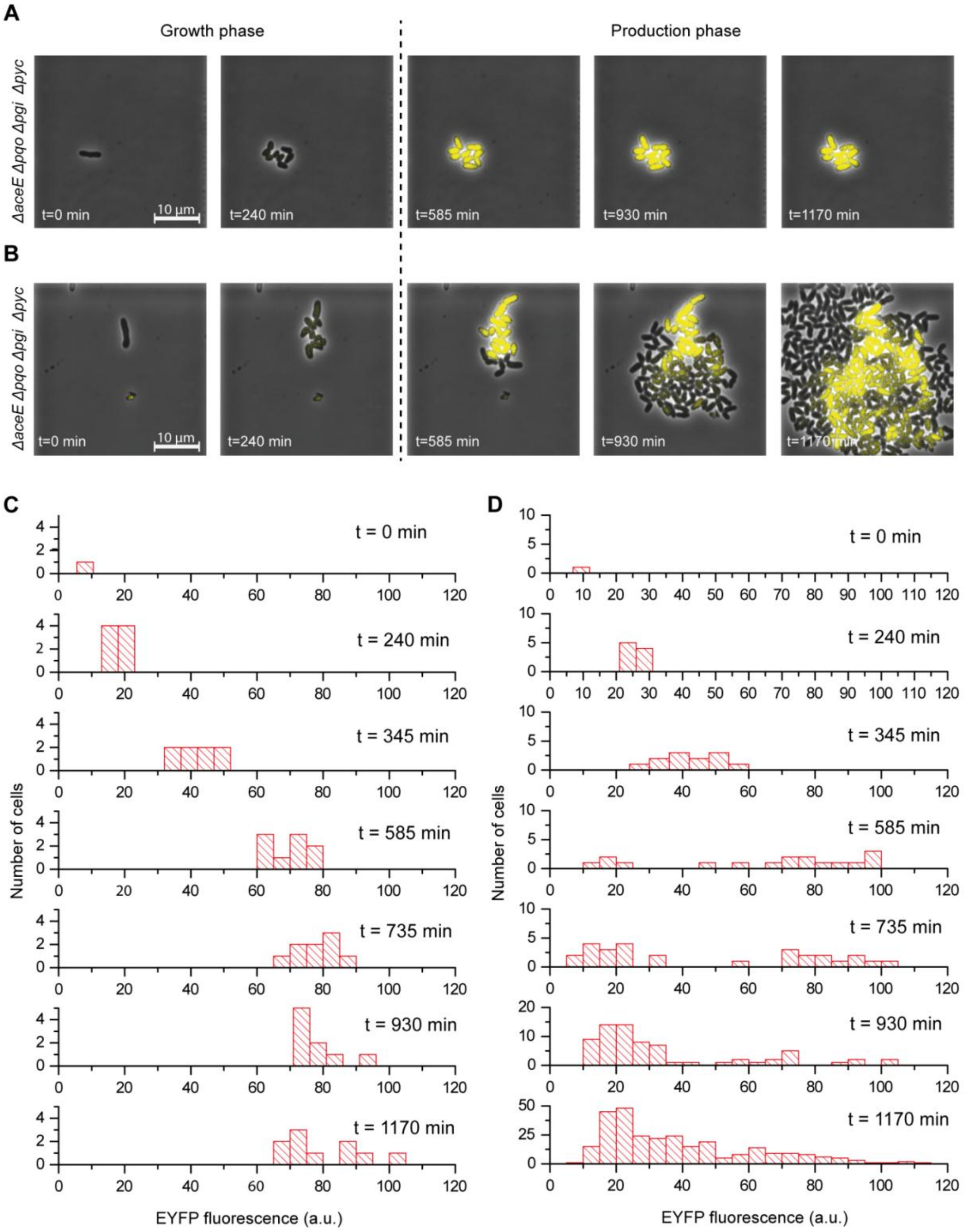

Figure S1. Phenotypic heterogeneity of the Δ*aceE* Δ*pqo* Δ*pgi* Δ*pyc* sensor strain upon switch from growth to production phase. (A) Microcolony showing transition to producing cells or **(B)** a mixture of growing and producing cells after medium switch (initiated after 240 min). In approximately 50% of the recorded colonies one or several single cells continued growth after medium switch. **(C, D)** Fluorescence histograms depicting single cell fluorescence to selected times during growth (0-240 min) and production phase (0-1200 min) of the microcolonies shown in A **(C)** and B **(D)**. Cultivation was performed in CGXII minimal medium containing 154 mM acetate, 222 mM glucose and 0.5% BHI during growth phase or 222 mM glucose and 0.5% BHI during production phase, respectively.

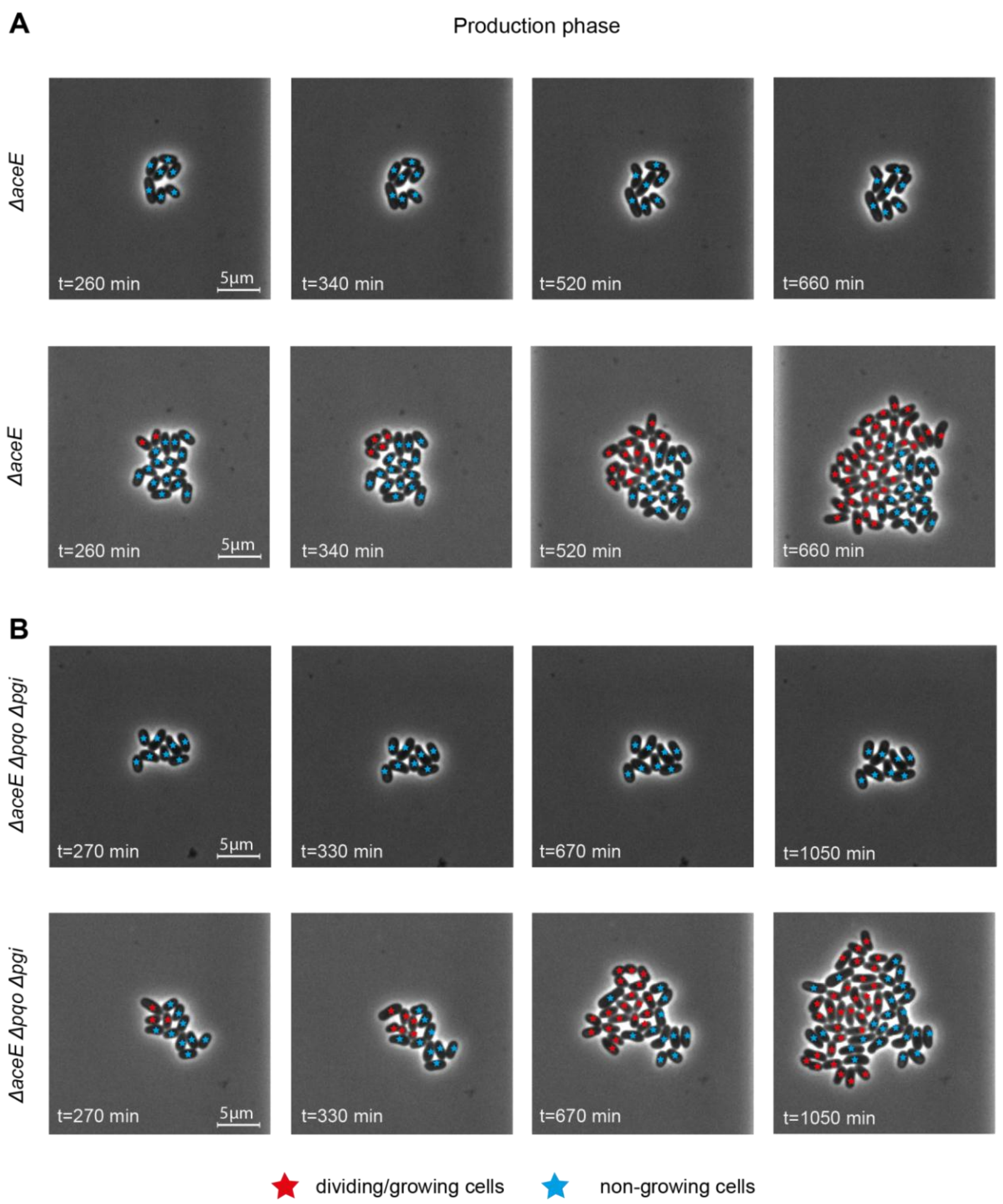

Figure S2. Phenotypic heterogeneity of Δ*aceE* and Δ*aceE* Δ*pqo* Δ*pgi* upon switch from growth to production phase. (A) Δ*aceE* microcolonies where all cells stopped growth (blue stars) upon transition to the production phase (upper row) or a mixture of growing (red stars) and non-growing cells (lower row) after initiation of the production phase. In approximately 50% of the recorded colonies one or several single cells continued growth after medium switch (initiated after 250 min). **(C)** Δ*aceE* Δ*pqo* Δ*pgi* microcolonies. In the upper row, all cells stopped growth whereas in the lower row a microcolony is shown were some cells continued growth after initiation of the production phase. In approximately 50% of the recorded colonies one or several single cells continued growth after medium switch (initiated after 250 min). These findings confirm that the phenotypic split shown in Figure 5 is not due to the presence of the Lrp-sensor. Cultivation was performed in CGXII minimal medium containing 154 mM acetate, 222 mM glucose and 0.5% BHI during growth phase or 222 mM glucose and 0.5% BHI during production phase, respectively.

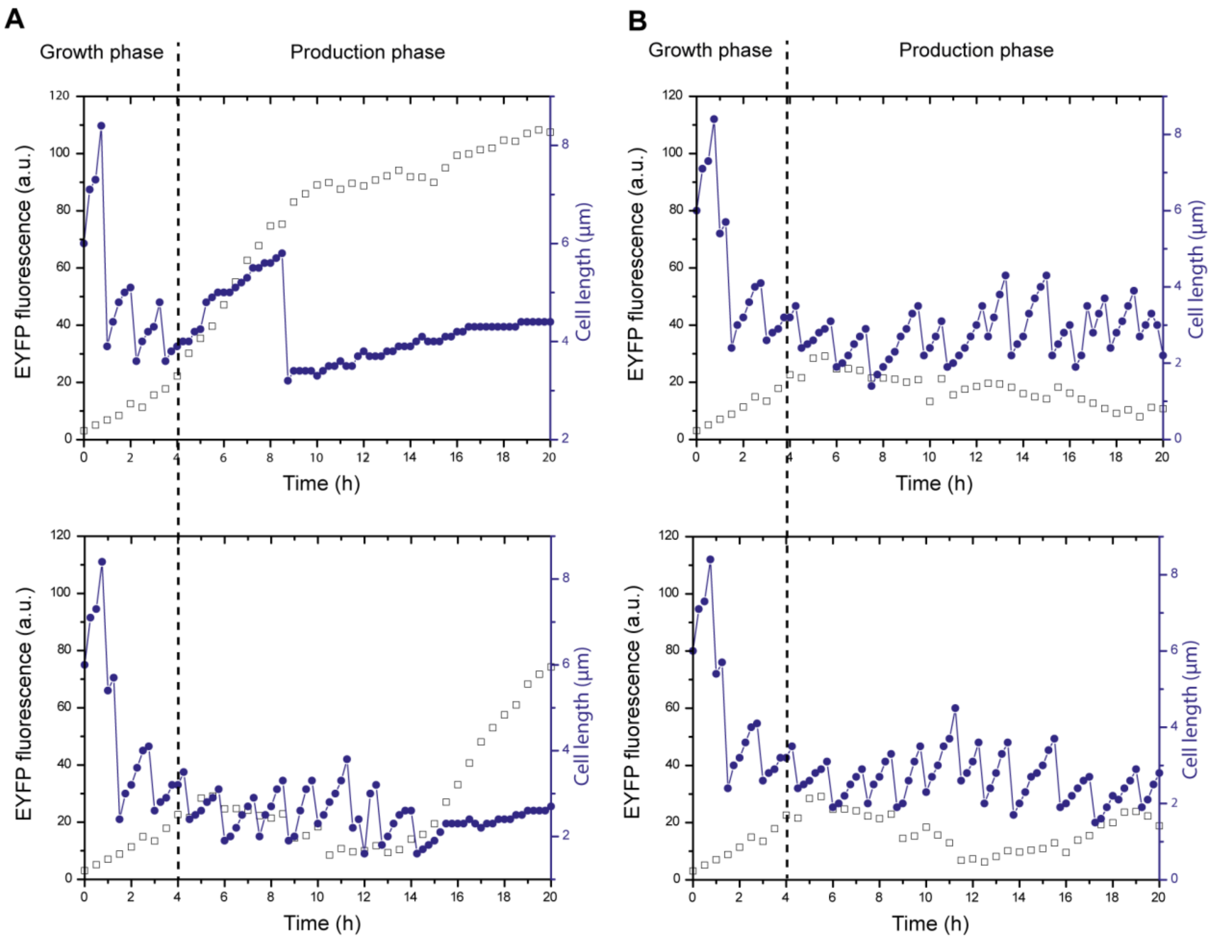

Figure S3. Single cell traces of the Δ*aceE* Δ*pqo* Δ*pgi* Δ*pyc* sensor strain upon switch from growth to production phase. (A) Single cell traces showing the switch from growth (cell length=blue line) to production (fluorescence=squares) after several cell divisions during production phase (t=8.5 h, t=15.0 h). **(B)** Single cell traces showing no switch from growth to production. Single cell traces are taken from the cultivation of Δ*aceE* Δ*pqo* Δ*pgi* Δ*pyc* sensor strain shown in Figure S1.
